# Relationship between left atrial appendage peak flow velocity and nonvalvular atrial fibrillation recurrence after cryoablation

**DOI:** 10.3389/fcvm.2023.1053102

**Published:** 2023-03-02

**Authors:** Long Wang, Yan Zhang, Wei Zhou, Jingjing Chen, Yongkang Li, Qian Tang, Bingxiu Chen, Huiling Zhang, Lucas Zellmer, Jin Chen, Zhangrong Chen, Wei Li, Xingde Liu, Haiyan Zhou

**Affiliations:** ^1^Department of Cardiology, The Affiliated Hospital of Guizhou Medical University, Guiyang, Guizhou, China; ^2^Department of Internal Medicine, Hennepin County Medical Center, Minneapolis, MN, United States; ^3^Department of Internal Medicine, School of Clinical Medicine, Guizhou Medical University, Guiyang, Guizhou, China

**Keywords:** left atrial appendage peak flow velocity, recurrence of nonvalvular atrial fibrillation, cryoballoon ablation, transesophageal echocardiography, corhort study

## Abstract

**Background:**

Previous studies revealed the connection between left atrial appendage peak flow velocity (LAA-PEV) and postoperative persistent atrial fibrillation (AF) recurrence. Yet, this association is not necessarily generalizable to persistent AF patients undergoing initial cryoballoon ablation due to current gaps in the literature.

**Methods:**

We prospectively studied 74 consecutive individuals with persistent atrial fibrillation undergoing a cryoballoon ablation for the first time between January 2018 and January 2020. Before ablation, LAA-PEV was documented by transesophageal echocardiography (TEE). Subsequently, demographic information and other clinical characteristics of these participants were collected. A 96-h continuous cardiac monitor was reviewed regularly for recurrence of atrial fibrillation. Cox proportional hazards regression was used to investigate LAA-PEV as well as other clinical characteristics as a predictor of AF recurrence.

**Results:**

Our study found that AF recurrences had lower LAA-PEV than those without AF recurrence. A nonlinear relationship between the LAA-PEV and AF recurrence was observed in this study, which had an inflection point of 34.9. Subgroup analysis of female participants showed that LAA-PEV had a positive correlation with AF recurrence [β = 0.8, 95% CI (0.7, 0.9), *p* < 0.05].

**Conclusion:**

A low LAA-PEV is related to recurrence of atrial fibrillation and may predict AF recurrence after initial cryoballoon ablation for persistent atrial fibrillation. This finding may help improve treatment and care strategies for patients with persistent atrial fibrillation.

## Introduction

Atrial fibrillation (AF) is the most common sustained cardiac arrhythmia, with high morbidity and mortality from stroke and thromboembolism (1). Guidelines recommend the use of antiarrhythmic drugs and catheter ablation to maintain of sinus rhythm in AF patients (2). Cryoballoon ablation is one of the most frequently used catheter ablation technologies which leads to cellular necrosis by tissue freezing and has shown promise in patients with atrial fibrillation (3). However, previous studies showed that recurrence after application of cryoballoon ablation within 1 year varied from 23.8 to 39.3% in nonvalvular AF (4). Hence, identifying reliable factors that affect AF recurrence before cryoballoon ablation may help improve treatment strategies and guide clinical practice.

Transoesophageal echocardiography (TEE) is an established component in the treatment of patients with nonvalvular AF used to screen for left atrial appendage (LAA) thrombi and allowing for earlier cardioversion (5, 6). Studies have reported that a LAA velocity < 40 cm/s could be used as a prognostic threshold for an increased risk of stroke and the presence of spontaneous echo contrast in AF patients (7). Velocities<20 cm/s could be used as a prognostic threshold for higher rates of thromboembolic events and detection of LAA thrombosis. So far, only a few studies have mentioned the relationship between left atrial appendage peak flow velocity (LAA-PEV) and persistent AF recurrence (8). However, these studies focused on the link between LAA-PEV and persistent AF recurrence after initial radiofrequency ablation (9), otherwise the correlation between LAA-PEV and paroxysmal AF recurrence after cryoballoon ablation is not established. The STOP Persistent AF Trial demonstrated cryoballoon ablation is safe and effective in treating patients with persistent AF. Investigating the connection between LAA-PEV and persistent AF recurrence is critical.

Our prospective cohort study was designed to investigate whether LAA-PEV is a predictor of persistent AF recurrence in patients undergoing an initial cryoballoon ablation. This study will give clinical data as well as fresh insights into the relationship between LAA-PEV and persistent AF recurrence undergoing an initial cryoballoon ablation.

## Materials and methods

### Study population

A prospective cohort clinical trial was performed and 74 consecutive patients with nonvalvular, persistent atrial fibrillation who underwent a first cryoballoon ablation were enrolled at the Affiliated Hospital of Guizhou Medical University between January 2018 and January 2020. The inclusion criteria were age older than 18 years and younger than 80 years, diagnosis of persistent AF and less than 1 year of persistent AF, and available echocardiographic data. We excluded patients with a history of cardiac surgery, congestive heart failure (CHF) [New York Heart Association (NYHA) ≥ III class], moderate to severe mitral regurgitation or stenosis, hepatopathy, hyperthyroidism, and atrial or ventricular thrombus from consideration. Additionally, all individuals were classified into three groups according to different LAA-PEV: low LAA-PEV group (18.2 cm/s-32 cm/s), medium LAA-PEV group (32 cm/s-44 cm/s), high LAA-PEV group (44 cm/s-82.5 cm/s). All procedures involving human subjects in this study were in compliance with the Declaration of Helsinki (updated 2013) (10). This clinical trial was approved by the Human Ethics Review Board of the affiliated Hospital of Guizhou Medical University, and informed consent was obtained from all patients.

### Measurement

Clinical and demographic information were collected, including age, sex, Body Mass Index (BMI), tobacco use, hypertension, prior history of diabetes mellitus, and current medications. Patients with nonvalvular AF were assigned a HAS-BLED score to assess the risk of major bleeding and a CHA2DS2-VASc score to assess the risk of thromboembolism. Additionally, the levels of LDL cholesterol (LDL-C) and HDL cholesterol (HDL-C) were tested by routine laboratory techniques.

### Transesophageal echocardiography

**Figure 1:**
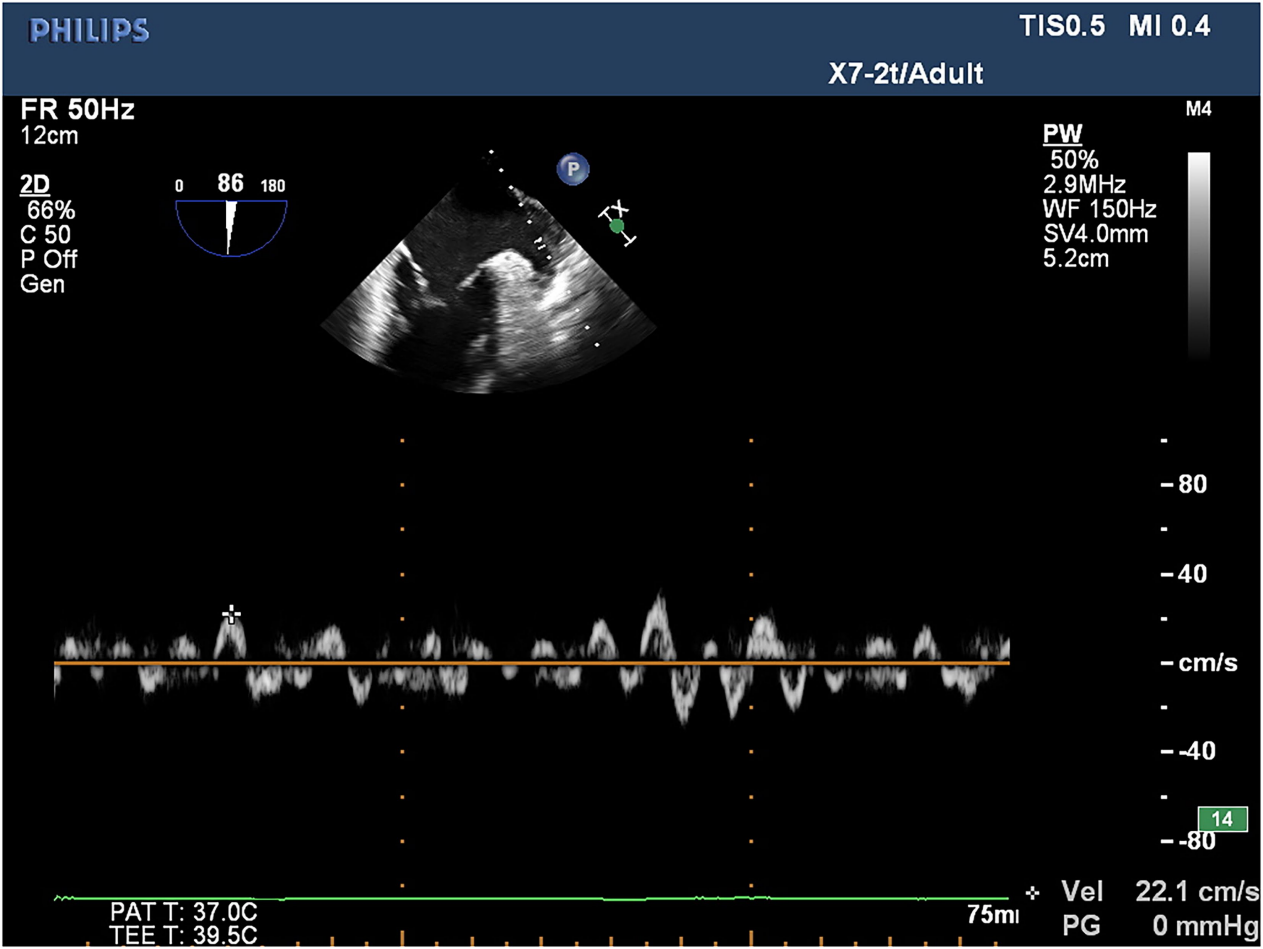
PW method was used to measure LAA-PEV under AF.

TEE was conducted utilizing a Philips iE33 color Doppler ultrasonic diagnostic instrument and quantitative analysis software QLab 8.1 for all patients with atrial fibrillation to assess for the presence of thrombosis within the left atrium and left atrial appendage within 24 h prior to cryoballoon catheter ablation. For TEE, the real-time three-dimensional transesophageal matrix probe (X7-2t) was utilized with athe frequency set to 2–7 MHz, while the LAA-PEV was measured by the pulsed wave doppler (PW) method. In patients in sinus rhythm, the flow spectrum of the LAA was a regular bidirectional waveform: positive waveform was an emptying wave, and negative waveform was a filling wave. In patients with AF, the flow spectrum of the LAA was an irregular serrated waveform, and the peak value of its positive waveform was selected as LAA-PEV (Figure 1).

### Cryoballoon catheter ablation procedure

**Figure 2:**
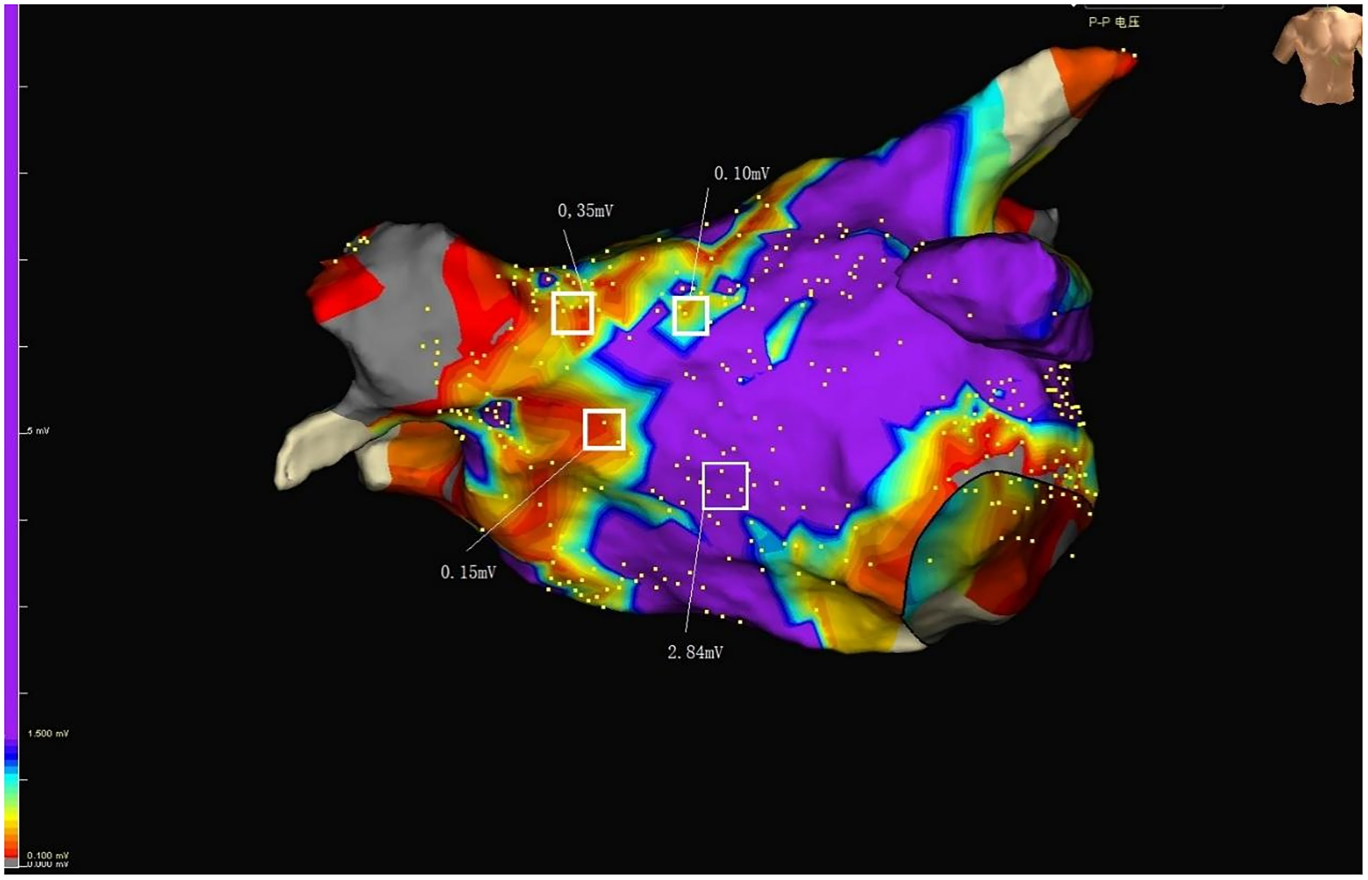
Purple represents normal voltage. Colors other than purple represent low voltage. RSPV, right superior pulmonary vein; RIPV, right inferior pulmonary vein; LSPV, left superior pulmonary vein; LIPV, left inferior pulmonary vein; MV, mitral valve; LAA, Left atrial appendage.

**Figure 3:**
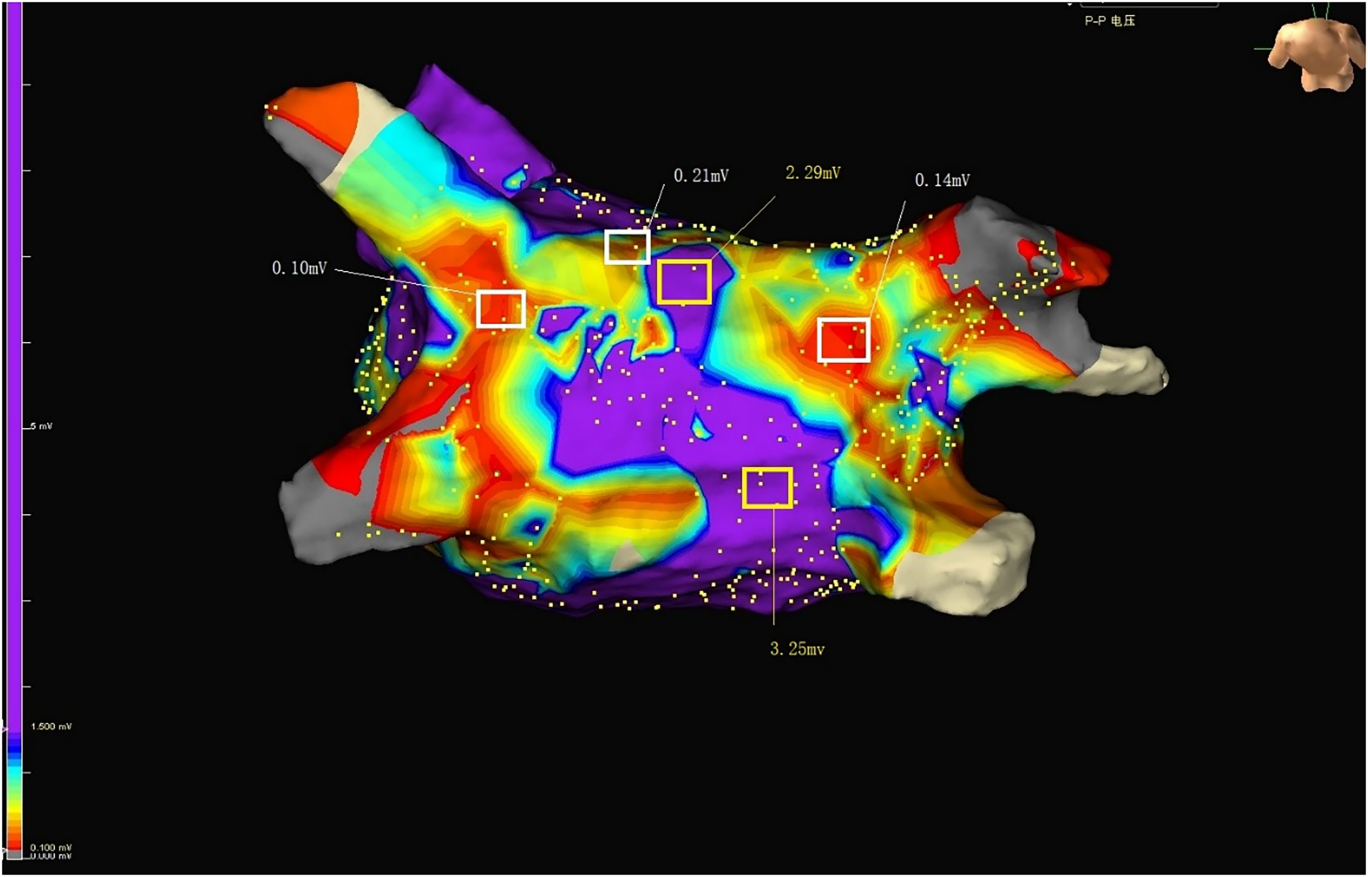
Purple represents normal voltage. Colors other than purple represent low voltage. RSPV, right superior pulmonary vein; RIPV, right inferior pulmonary vein; LSPV, left superior pulmonary vein; LIPV, left inferior pulmonary vein; LAA, Left atrial appendage.

Cryoballoon ablation of AF was performed as previously described (11). The EnSite Precision system (Abbott EP, United States) was used to guide the pulmonary vein isolation (PVI) process. The main procedures were listed as following: First, a single transseptal puncture was performed using an RF needle (Abbott EP, United States). Second, using the EnSite Percision mapping system, the three-dimensional (3D) structure of the LA and pulmonary vein (PV) trunks was reassembled using a circular diagnostic mapping catheter (Achieve Mapping Catheter, Medtronic). Third, under sinus rhythm, the WorkMateTM ClarisTM electrophysiological recording system was used to statistically analyze the local bipolar voltage at each point in the LA, and the overall average value of the bipolar voltage at each point in the LA was calculated. A local bipolar voltage >0.5 mV in the LA was defined as normal voltage, while a bipolar voltage <0.5 mV was defined as a low voltage area (Figures 2, 3). To facilitate recording and description, the LA was divided into anterior, roof, and posterior anatomical regions. Subsequently, a transseptal puncture and an over-the-wire delivery approach was used to deliver a second-generation 28-mm cryoballoon (Arctic Front Advance Cardiac Cryoablation Catheter, Medtronic). Then, balloons were placed on each PV. Mapping catheters to confirm acute venous occlusion can be achieved by using dedicated lumens to detect inlet (and outlet) obstruction. All patients with atrial fibrillation must achieve PVI. Patients were treated with systemic anticoagulation therapy for at least 3 months after discharge. Re-ablation and application of AAD were allowed 90 days after surgery. After a 90-day ‘blanking period’, all AADs except calcium-channel blockers and beta-blockers were discontinued, and repeat ablations were defined as failure of the primary endpoint.

### AF recurrence assessment and follow-up

In a 3 month post-ablation ‘blanking period’, anti-arrhythmic medications were continued with no contraindications. Anticoagulation with warfarin sodium or DOACs (dabigatran and rivaroxaban) was recommended for at least 3 months for patients without contraindications. All patients with nonvalvular atrial fibrillation completed follow-up at 1, 3, 6, 12, and 18 months with standard 12-lead electrocardiograms (ECGs) and 96-h continuous cardiac monitoring. After the 3-month blanking period, the AF recurrence was defined as atrial flutter/AF/tachycardia with a duration >30 s and registered by an ECG or other device (12). The clinical follow-up and data were separately analyzed by two electrophysiologists. In this study, the time point for recurrence assessment was set at 12 months after ablation. If the follow-up time was less than 12 months, the actual follow-up time was recorded. All patients had a follow-up time of at least 6 months.

### Statistical analysis

If continuous variables were normally distributed as assessed according to the Kolmogorov–Smirnov test, they were summarized with means ± SDs and compared with independent samples *t*-tests. Otherwise, variables without normal distribution were summarized with medians and quartiles or ranges and compared with Mann–Whitney *U*-tests. To investigate whether LAA-PEV is associated with postoperative nonvalvular AF recurrence, they were first divided into three groups based on LAA-PEV, and then a weighted chi-square test or a weighted linear regression model was used to calculate the difference between the ternary arrays. Next, weighted univariate and multiple linear regression models were employed to determine the linear relationship between LAA-PEV and the postoperative nonvalvular AF recurrence. Three statistical models were constructed: model I, no adjustment covariates; model II, adjusted only based on social demographic data; and model III, shown in Table 1. Moreover, a stratified Cox proportional hazards model was utilized to perform the subgroup analyses. The adjusted test for subgroup effects used interaction terms between subgroup measures, followed by the likelihood ratio test. The statistical software package R (^1^The R Foundation) was used to conduct all of the analyses. A *p* value <0 0.05 (two-tailed) was considered statistically significant.

**Table 1:** Results of multivariate analysis using non-adjusted and adjusted cox regression models.

Exposure	Crude model (HR, 95%CI, *p*)	Minimally adjusted model (HR, 95%CI, *p*)	Fully adjusted model (HR, 95%CI, *p*)
LAA-PEV	0.8 (0.7, 0.9) <0.001	0.7 (0.6, 0.9) <0.001	0.8 (0.7, 1.0) 0.011
LAA-PEV (Ternary)			
Low (<32 cm/s)	1	1	1
Medium (≥32 cm/s, <44 cm/s)	0.0 (0.0, 0.1) <0.001	0.0 (0.0, 0.1) <0.001	0.0 (0.0, 0.2) 0.004
High (≥44 cm/s)	0.0 (0.0, 0.0) <0.001	0.0 (0.0, 0.0) <0.001	0.0 (0.0, 0.3) 0.010

Crude model: we did not adjust other covariants.

Minimally adjusted model: we adjusted age, sex, BMI, LDL, HDL.

Fully adjusted model: we adjusted age, sex, BMI, LDL, HDL, LAAPD, LVEF, the percentage of LVAs.

HR, hazard ratio; CI, confidence interval; Ref, reference.

## Results

### Baseline characteristics

We enrolled 74 consecutive patients with nonvalvular AF who underwent a first cryoballoon ablation and then compared the distribution of demographic information and clinical characteristics in different LAA-PEV groups (Table 2). There was no statistically significant difference in age, sex, BMI, HDL-c, LDL-c, blood pressure, smoking status, alcohol consumption, history of hypertension, or Diabetes mellitus. Compared with LAA-PEV high and medium groups, patients in LAA-PEV low group had a significantly higher AF recurrence.

**Table 2:** Baseline characteristics of patients with variable left atrial appendage peak flow velocity.

Characteristics	Low	Medium	High	*p*-value	*p*-value*
*N*	25	24	25		
Left atrial appendage peak flow velocity (cm/s)	24.5 ± 3.8	38.8 ± 4.1	58.8 ± 11.4	<0.001	<0.001
Age	66.6 ± 5.8	61.5 ± 7.8	64.4 ± 9.2	0.075	0.065
Male, *n* (%)	9 (36.0%)	11 (45.8%)	12 (48.0%)	0.66	
Heart rate	99.3 ± 20.0	95.1 ± 26.1	87.8 ± 21.5	0.196	0.113
BMI	23.6 ± 3.8	23.6 ± 3.1	23.9 ± 3.4	0.95	0.943
LDL	1.9 ± 0.7	2.1 ± 0.6	2.3 ± 0.9	0.337	0.361
HDL	1.3 ± 0.4	1.1 ± 0.3	1.1 ± 0.3	0.223	0.104
Systolic pressure	128.2 ± 25.1	129.0 ± 19.8	129.9 ± 20.1	0.965	0.961
Diastolic pressure	83.6 ± 18.5	82.2 ± 18.1	80.4 ± 18.1	0.82	0.941
Hypertension, *n* (%)	13 (52.0%)	14 (58.3%)	12 (48.0%)	0.766	
Diabetes mellitus, *n* (%)	2 (8.0%)	5 (20.8%)	3 (12.0%)	0.407	–
Smoking, *n* (%)	7 (28.0%)	3 (12.5%)	9 (36.0%)	0.161	–
Heavy alcohol intake (>60 mL/day)	9 (36.0%)	4 (16.7%)	10 (40.0%)	0.17	–
AF recurrence	23 (92.0%)	2 (8.3%)	1 (4.0%)	<0.001	–
Medications, *n* (%)					
ACEI/ARB	4 (16.0%)	6 (25.0%)	0 (0.0%)	0.034	–
β-blockers	11 (44.0%)	8 (33.3%)	7 (28.0%)	0.483	–
Calcium Antagonists	8 (32.0%)	10 (41.7%)	5 (20.0%)	0.259	–
NOAC	24 (96.0%)	22 (91.7%)	25 (100.0%)	0.335	0.315
CHA2DS2-VASc score	2.9 ± 0.7	1.9 ± 1.2	1.9 ± 1.1	<0.001	0.001
HAS-BLED score	1.2 ± 0.8	0.7 ± 0.8	0.8 ± 0.6	0.072	0.053

### Correlation between clinical characteristics and AF recurrence

In order to explore correlation between clinical characteristics and AF relapse, univariate analyses were conducted (Table 3). We found that the left atrium anterior and posterior diameter (LAAPD) (1.3, 1.2–1.5), the percentage of low-voltage areas (LVAs) (1.2, 1.1–1.3), and CHA2DS2-VASc score (2.3, 1.3–4.1) were associated with an increased risk of nonvalvular AF recurrence. On the contrary, univariate analysis showed that LAA-PEV (0.8, 0.7–0.9) and LVEF (0.8, 0.7–0.9) were correlated with a lower recurrence of nonvalvular AF.

**Table 3:** Results of univariate analysis.

	Statistics	β (95%CI), *p* value
Age	64.2 ± 7.9	1.0 (1.0, 1.1) 0.193
Sex		
Male	32 (43.2%)	1
Female	42 (56.8%)	1.7 (0.6, 4.7) 0.272
Heart rate	94.1 ± 22.8	1.0 (1.0, 1.0) 0.319
BMI	23.7 ± 3.4	1.0 (0.9, 1.1) 0.883
LDL	2.1 ± 0.8	0.6 (0.3, 1.3) 0.208
HDL	1.2 ± 0.3	2.4 (0.6, 10.2) 0.235
Systolic pressure	129.1 ± 21.5	1.0 (1.0, 1.0) 0.841
Diastolic pressure	82.1 ± 18.0	1.0 (1.0, 1.0) 0.281
Hypertension		
No	35 (47.3%)	1
Yes	39 (52.7%)	1.1 (0.4, 2.8) 0.885
Diabetes mellitus		
No	64 (86.5%)	1
Yes	10 (13.5%)	0.8 (0.2, 3.2) 0.715
Current.Smoker		
No	55 (74.3%)	1
Yes	19 (25.7%)	1.1 (0.4, 3.3) 0.857
Heavy Alcohol intake (>60 ml/day)		
No	51 (68.9%)	1
Yes	23 (31.1%)	1.3 (0.5, 3.6) 0.629
LAAPD, mm	40.2 ± 6.5	1.3 (1.2, 1.5) <0.001
LAA-PEV (cm/s)	40.7 ± 16.0	0.8 (0.7, 0.9) <0.001
LVEF	55.9 ± 10.5	0.8 (0.7, 0.9) <0.001
The percentage of LVAs	5.7 ± 8.9	1.2 (1.1, 1.3) <0.001
CHA2DS2-VASc score	2.3 ± 1.1	2.3 (1.3, 4.1) 0.003
HAS-BLED score	0.9 ± 0.8	1.6 (0.9, 3.1) 0.131

### Relationship between the LAA-PEV and postoperative nonvalvular AF recurrence

Cox regression models (non-adjusted and adjusted model) were used to examine whether LAA-PEV was associated with nonvalvular AF recurrence (Table 1). In a non-adjusted model, for each 1 cm/s increase in LAA-PEV, the risk of nonvalvular AF recurrence was decreased by 20% (HR = 0.8, 95% confidence interval (CI): 0.7–0.9, *p* < 0.001). In a minimally adjusted model (adjusted age, sex, BMI, LDL, HDL), the risk of nonvalvular AF recurrence was decreased by 30% (HR = 0.7, 95%CI: 0.6–0.9, *p* < 0.001). In a fully adjusted model (adjusted sex, BMI, age, LDL, HDL, LAAPD, LVEF, the percentage of LVAs), the trend of HR was not obviously altered (HR = 0.8, 95%CI: 0.7–1.0, *p* = 0.011). HR trends under different adjustment strategies indicated that LAA-PEV was an independent protective factor for non-valvular atrial fibrillation recurrence, and the results were consistent. Additionally, we included LAA-PEV as a categorical (Ternary) variable for sensitivity analysis. Similar trends were also observed (*p* for trend was <0.001).

### Analyses of nonlinear relationships and threshold effects of LAA-PEV and postoperative nonvalvular AF recurrence

**Figure 4:**
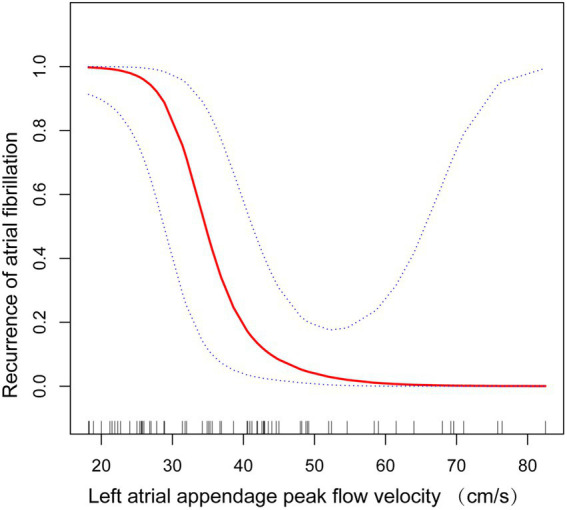
Non-linear correlation between left atrial appendage peak flow velocity and recurrence of atrial fibrillation in stratified analysis.

Since LAA-PEV is a continuous variable, we explored the possibility of a non-linear association between the LAA-PEV and nonvalvular atrial fibrillation recurrence. In our study (Figure 4), a non-linear association between the LAA-PEV and nonvalvular AF recurrence was found (after adjusting for sex, BMI, alcoholic consumption, smoking status, hypertension history, diabetes history, the level of LDL and HDL). An inflection point of 34.9 was calculated using a two-part linear regression model. On the left of inflection point, the effect size, 95% CI and value of p were 0.6, 0.4–0.8, and *p* = 0.001, respectively. However, no relationship was found between LAA-PEV and the recurrence of nonvalvular atrial fibrillation on the right of inflection point (0.9, 0.8–1.1, 0.220) (Table 4).

**Table 4:** Nonlinearity explanation on LAA-PEV and AF recurrence using two-piece wise linear model.

Inflection point of LAA-PEV (Per 0.1 change)	Effect size (95% CIs)	*p*-value
<34.9	0.6(0.4, 0.8)	0.001
≥34.9	0.9(0.8, 1.1)	0.22
*p* for log likelihood ratio test		0.039

Adjusted for sex, BMI, smoking status, alcoholic consumption, hypertension history, diabetes history, the level of LDL and HDL.

### Subgroup analyses

In our study, interactions were seen between clinical characteristics and AF recurrence (Table 5). We observed that the interaction test for sex (*p* for interaction = 0.0067) and the percentage of LVAs (*p* for interaction = 0.0028) was significant. The test for interactions were not statistically significant for hypertension, LVEF, BMI, or heart rate (p for interaction = 0.5672, 0.7466, 0.3614, 0.3300 respectively). The interactions between LAA-PEV and sex were observed in subgroup analyses. In male AF patients, HR was 0.0 (0.0–INF). In female AF patients, HR was 0.8(0.7–0.9). HR values between male and female had significant differences (detected by log-likelihood ratio test). Additionally, an interaction between LAA-PEV and the percentage of LVAs was observed. In AF patients with the middle percentage of LVAs, HR was 0.8 (0.7–0.9), while in AF patients with high percentage of LVAs, HR was 0.0(0.0–170.1). There is a significant difference between AF patients with the middle percentage of LVAs and high percentage of LVAs.

**Table 5:** The effect size of LAA-PEV on AF recurrences in subgroups.

Characteristic	Numbers of participants	Effect size (95%CI)	*p* value	*p* (interaction)
Sex				0.0067**
Male	32	0.0 (0.0, Inf)	0.9953	
Female	42	0.8 (0.7, 0.9)	0.0008***	
Hypertension				0.5672
No	35	0.7 (0.5, 0.9)	0.0069**	
Yes	39	0.8 (0.7, 0.9)	0.0013**	
LAAPD (Ternary)				0.0127*
Low (<36 mm)	21	0.8 (0.5, 1.2)	0.2958	
Middle (≥36 mm, <43 mm)	28	0.8 (0.7, 1.0)	0.0175*	
High (≥43 mm)	25	0.0 (0.0, Inf)	0.9954	
LVEF (Ternary)				0.7466
Low (<50)	24	0.7 (0.5, 1.0)	0.0270*	
Middle (≥50, <60)	21	0.8 (0.6, 1.0)	0.0194*	
High (≥60)	29	0.8 (0.5, 1.1)	0.1688	
The percentage of LVAs				0.0028**
<3%	45	0.8 (0.7, 0.9)	0.0039**	
>3%	29	0.0 (0.0, 170.1)	0.3397	
BMI (Ternary)				0.3614
Low (<22)	25	0.7(0.5, 1.0)	0.0309*	
Middle (≥22, <25)	24	0.6 (0.4, 1.0)	0.0312*	
High (≥25)	25	0.8 (0.7, 1.0)	0.0213*	
Heart rate (Ternary)				0.33
Low (<80)	25	0.8 (0.7, 1.0)	0.0125*	
Middle (≥80, <100)	24	0.7 (0.6, 1.0)	0.0248*	
High (>100)	25	0.6 (0.4, 0.9)	0.0223*	

## Discussion

This prospective cohort study enrolled 74 patients with persistent AF, which revealed a consistent correlation between the LAA-PEV and the postoperative nonvalvular AF recurrence after adjustment of potential confounders. Additionally, a non-linear association between the LAA-PEV and risk of AF recurrence was found. The linear decrease between recurrence risk and the LAA-PEV reached a peak velocity of 34.9 cm/s (saturation effect), and an elevation in velocity above 34.9 cm/s did not increase the risk of AF recurrence.

LAA-PEV reflects left atrial contractile function (13). The relationship between reduced LAA-PEV and increased risk of AF recurrence after catheter ablation has previously been observed. By studying 53 patients with persistent AF undergoing an initial catheter ablation, Takashi Kanda et al. reported that patients with AF recurrence had lower LAA-PEV than those without AF recurrence (14). Wentao Yang et al. reported that low LAA-PEV is correlated with AF recurrence, and it can predict AF recurrence following the initial radiofrequency catheter ablation for persistent AF (8). However, most of these studies investigated patients who underwent radiofrequency-based ablation. The Food and Drug Administration (FDA) approved cryoballoon catheter ablation to treat recurrent, symptomatic, drug-refractory paroxysmal AF (15), and persistent atrial fibrillation (16). It is reported that patients with persistent AF recurrence treated with an initial cryoballoon catheter ablation at 1-year varied from 23.8 to 39.3% (17). Research performed by Demet Menekşe Gerede et al. shows that the presence of low LAA-PEV and low peak PV systolic wave velocity can predict the recurrence of paroxysmal AF after cryoablation (18). Their findings are consistent with ours. Our findings indicate that low LAA-PEV is associated with AF recurrence after cryoballoon catheter ablation for persistent AF. Furthermore, we established a non-linear relationship between low LAA-PEV and AF recurrence with an inflection point of 34.9 cm/s. In the present study, we used sex, LAAPD, LVEF, percentage of LVAs, BMI, heart rate, and hypertension history as stratification variables. Furthermore, we found that low LAA-PEV is associated with AF recurrence after cryoballoon catheter ablation for persistent AF, especially in females.

Our research has several benefits. First, our study is a prospective cohort study with strict inclusion and exclusion criteria compared with the previous studies. Several potential confounders were excluded using the described adjustment strategy. Second, we found the changes in the effect size of LAA-PEV as continuous variables and categorical variables and tested the value of p for trend using LAA-PEV as a categorical variable, which showed convincing results. Third, previous studies observed that lower LAA-PEV have a significant effect on AF recurrence (19). Fourth, a generalized additive model was utilized to study the nonlinear correlations. This approach has several benefits as the model can support nonparametric smoothing and fit regression splines to the data; thus helping us better understand the true relationship between predictors and outcomes (17). Fifth, since observational studies appear to be confounded, rigorous statistical adjustments were made to reduce confounding.

### Limitations

Our study has some limitations. First, our data only come from Chinese patients, and the research scope is limited. Secondly, the study was conducted at a single site with a small sample size and therefore leading to selection bias. Third, we focused on patients with persistent AF, so our results can not be applied to patients with paroxysmal AF.

## Conclusion

Our research reported a non-linear association between LAA-PEV and AF recurrence and can predict AF recurrence following an initial cryoablation for persistent AF. AF recurrence following an initial cryoablation for persistent AF increases by 40% for every 1 cm/s decrease in LAA-PEV below the velocity of 34.9 cm/s. Our findings may help improve treatment and care strategies of individuals with persistent AF undergoing an initial cryoballoon ablation.

## Data availability statement

The original contributions presented in the study are included in the article/Supplementary material, further inquiries can be directed to the corresponding authors.

## Ethics statement

The studies involving human participants were reviewed and approved by affiliated Hospital of Guizhou Medical University. The patients/participants provided their written informed consent to participate in this study.

## Author contributions

HZho and XL: conception and design and administrative support. LW, YZ, YL, and WZ: provision of study materials. JC, ZC, and JC: collection and assembly of data. BC, QT, and HZha: data analysis and interpretation. All authors: manuscript writing and final approval of manuscript.

## Funding

This work was supported by the National Natural Science Foundation of China (81904319, 82060855, 82260987, 82160086, 81960085), the Science and Technology Fund of Guizhou Provincial Health Department (qiankehejichu-ZK[2022]zhongdian043, qiankehepingtairencai-GCC[2022]040-1, qiankehezhicheng[2019]Y280), the Fund of Guiyang Science and Technology Department (zhukehetong[2021]43-6), the Health and Family Planning Commission of Guizhou Province (gzwjkj2021-106, gzwjkj2020-2-001), the Fund of the Affiliated Hospital of Guizhou Medical University (gyfybsky-2021-33, 2021-GMHCT-020).

## Conflict of interest

The authors declare that the research was conducted in the absence of any commercial or financial relationships that could be construed as a potential conflict of interest.

## Publisher’s note

All claims expressed in this article are solely those of the authors and do not necessarily represent those of their affiliated organizations, or those of the publisher, the editors and the reviewers. Any product that may be evaluated in this article, or claim that may be made by its manufacturer, is not guaranteed or endorsed by the publisher.

## Footnotes

^1^ http://www.R-project.org
